# Acceptance of selective contracting: the role of trust in the health insurer

**DOI:** 10.1186/1472-6963-13-375

**Published:** 2013-10-02

**Authors:** Romy E Bes, Sonja Wendel, Emile C Curfs, Peter P Groenewegen, Judith D de Jong

**Affiliations:** 1 NIVEL (Netherlands institute for health services research), Otterstraat 118 – 124, 3513, CR Utrecht, The Netherlands; 2 Open University, Valkenburgerweg 177, 6419, AT Heerlen, The Netherlands

**Keywords:** Health insurer, Trust, Managed competition, Selective contracting

## Abstract

**Background:**

In a demand oriented health care system based on managed competition, health insurers have incentives to become prudent buyers of care on behalf of their enrolees. They are allowed to selectively contract care providers. This is supposed to stimulate competition between care providers and both increase the quality of care and contain costs in the health care system. However, health insurers are reluctant to implement selective contracting; they believe their enrolees will not accept this. One reason, insurers believe, is that enrolees do not trust their health insurer. However, this has never been studied. This paper aims to study the role played by enrolees’ trust in the health insurer on their acceptance of selective contracting.

**Methods:**

An online survey was conducted among 4,422 people insured through a large Dutch health insurance company. Trust in the health insurer, trust in the purchasing strategy of the health insurer and acceptance of selective contracting were measured using multiple item scales. A regression model was constructed to analyse the results.

**Results:**

Trust in the health insurer turned out to be an important prerequisite for the acceptance of selective contracting among their enrolees. The association of trust in the purchasing strategy of the health insurer with acceptance of selective contracting is stronger for older people than younger people. Furthermore, it was found that men and healthier people accepted selective contracting by their health insurer more readily. This was also true for younger people with a low level of trust in their health insurer.

**Conclusion:**

This study provides insight into factors that influence people’s acceptance of selective contracting by their health insurer. This may help health insurers to implement selective contracting in a way their enrolees will accept and, thus, help systems of managed competition to develop.

## Background

In recent last decades, health care reforms have been implemented in several countries. A general aspect of most of these reforms is a shift from a supply-oriented system to a demand-oriented one. In several countries these reforms were based on introducing managed competition, for instance in Germany, Switzerland and the United States [1-3]. In a system of managed competition, health insurers play an important role. The idea is that they become prudent buyers of care on behalf of their enrolees. Health insurers can negotiate contracts with care providers and in some forms of managed competition health insurers are allowed to selectively contract care providers. This is supposed to increase competition between care providers and in turn increase the quality of care and contain the costs of health care. However, when health insurers choose to selectively contract with care providers, it is important that they channel their enrolees towards these contracted care providers. Competition between care providers will only happen when care providers fear they will lose a share of the market if they are not contracted by the insurers [4-7]. In addition, in a system of managed competition there is also competition between health insurers. People have the option of switching insurers if they are not satisfied or can get a better offer elsewhere. Selective contracting has an important implication for enrolees. It means that their freedom of choice of care provider is restricted to those care providers selected by their health insurer. There are two forms of selective contracting. One is based on 'preferred’ provider networks and means that enrolees are also allowed to use providers that are not contracted by their health insurer, but this care will only partially be reimbursed. The other form is 'exclusive’ , which means that enrolees will receive no reimbursement at all when they to go to a care provider that is not contracted by their insurer. The premium for restrictive health plans, in which care providers are selectively contracted by the health insurer, is usually lower, because the health insurer can purchase greater volumes of care from fewer care providers at a lower unit price.

Health insurers, however, are still reluctant to implement selective contracting in the Netherlands, even though a system of managed competition was implemented in 2006 (the key points of the Dutch health care system are listed in Table 1). This is because they fear they will lose their enrolees, since both provider and consumer organisations are critical about restrictions to the freedom of choice of provider [8]. Boonen has interviewed large health insurance companies in the Netherlands about why they are reluctant to implement selective contracting [9]. The most important reason given by the insurers is that they expect that their enrolees do not trust them to act as good purchasing agents on their behalf [9]. Other literature also points out that trust in the health insurer could play a key role in whether or not enrolees accept selective contracting [10-13]. Yet, trust may not be the only factor, there could be other factors that influence in how far enrolees accept selective contracting by their health insurer, such as reluctance to change and the importance of autonomy in the choice of care provider. However, we specifically focus in this article on the trust of enrolees in their health insurer, because this relationship has not yet been studied. No research has yet been done to see whether enrolees who have more trust in their health insurer, are more accepting of selective contracting by their health insurer. Neither has it been made clear what it is that people trust which influences their acceptance of selective contracting. Is it their trust in their health insurer in general, or is it their trust in the way their health insurer selects care providers? Additionally, we will investigate to what extent enrolees’ characteristics play a role in the relationship between the trust in their health insurer and their acceptance of selective contracting. Therefore, the following research question will be answered in this paper: What role does enrolees’ trust in their health insurer play in their acceptance of selective contracting? This question is relevant for all countries where a system of managed competition is implemented or will be implemented in the future.

**Table 1:** Key elements of the Dutch health care system (From: Enthoven and Van de Ven, 2007)

1	Mandatory basic health insurance for everyone, purchased through private insurance companies
2	Annual consumer choice of insurer and insurance products
3	Open enrolment and community rating
4	Premium subsidies for elderly people and those at high risk of disease, through a risk-equalisation system
5	Mandatory deductible of €165 per person per year*
6	Voluntary deductible up to €500 per person per year
7	Insurers allowed to sell other types of insurance (e.g., supplementary insurance)
8	Insurers intended to be the prudent buyers of care on behalf of their members
9	General practitioners to serve as gatekeepers
10	Insurers permitted to contract selectively with doctors and hospitals
11	Health maintenance organisations and preferred provider arrangements allowed
12	In transition toward managed competition

*The government assesses the amount of the mandatory deductible every year. When this survey was conducted (end of 2010) it was €165.

### Trust

Trust is very important in health care [14-17], not only between patients and physicians, but also between patients and medical institutions such as hospitals and health insurers. Studies have found that trust has the same functional attributes as satisfaction. However, in contrast to satisfaction, which is an assessment of past events, trust is a forward-looking evaluation of an on-going relationship. Factors that generally underlie trust include fidelity, competence, honesty, confidentiality and global trust [17].

Trust is closely related to risk, which is derived from the uncertainty of the trustor regarding the motives, intentions and future actions of the trustee [14].

Theory states that if there is no uncertainty, no trust is needed and the higher the initial perception of risk, then the higher the trust needed to facilitate transactions [18]. Furthermore, trust is shown to reduce the perceived risk [18,19]. When trusting someone, you allow yourself to be vulnerable by running the risk that that person will exploit you [14]. We define trust according to definitions based on the work of Gilson and Hall et al. as the optimistic acceptance of a vulnerable situation where the trustor believes that the trustee has his best interests at heart [14,17].

Trust has previously been researched in the context of health insurers. There, results show, for instance, that trust in the health insurer correlates positively with a lower desire to switch insurers and fewer disputes with the insurer [17,20,21]. Trust is also related to enrolees’ overall assessment of their health insurer [22].

### Hypotheses

As has been mentioned, the literature implies that trust in the health insurer could play an important role in the willingness of enrolees to let their health insurer select care providers for them [9-11,13]. This is consistent with the literature on trust and risk. Enrolees do not know what care they will need in the future and neither do they have experience with all the care providers their health insurer contracts with. Because of this uncertainty or risk, they need to trust that their health insurer will have their best interests at heart. Therefore, we expect that trust in the health insurer is an important prerequisite for their enrolees’ acceptance of selective contracting.

H1: Enrolees who have more trust in their health insurer will be more accepting of selective contracting by their health insurer.

The perceived risk of enrolling in a health insurance policy with limited choice may be greater for less healthy people, because they are more likely to need care, now and in the near future. Since trust reduces perceived risk [18,19], we expect that, compared to healthy enrolees, less healthy ones need to have more trust in their health insurer in order to accept selective contracting. Therefore, we expect that the relationship between trust in the health insurer and acceptance of selective contracting will differ between healthy and unhealthy enrolees.

H2: The role of trust in the acceptance of selective contracting is stronger for unhealthy enrolees compared to healthy enrolees.

The same goes for older people, who are also more likely to need care in the near future. Therefore, they take a greater risk than younger people do when they agree to selective contracting by their health insurer. Hence, we expect that older people need to have more trust in their health insurer in order to accept selective contracting. Thus, we expect that the relationship between trust and openness to selective contracting will differ with age.

H3: The role of trust in the acceptance of selective contracting is stronger for older enrolees than for younger ones.

Women are more risk averse than men [23]. Thus, they may perceive greater risks in agreeing to selective contracting than men. Therefore, we expect that women need to have more trust in their health insurer when they agree to selective contracting.

H4: The role of trust in the acceptance of selective contracting is stronger for women than for men.

We also expect health, age and gender to be directly associated with the acceptance of selective contracting by the health insurer. This expectation is based on literature showing that young and healthy people more often choose cheaper health policies with restrictions on the range of providers than older people and people with health problems [24-26]. The reason for this may be that young and healthy people use less care and are therefore less concerned about their freedom of choice of provider. This leads to the expectation that younger and healthier enrolees will be more open to selective contracting than older and less healthy ones. Another reason for less healthy enrolees to be more reluctant to accept selective contracting can be that they have more experience with receiving health care and may have a stronger relationship with one or more of their care providers. They may be afraid of losing these regular providers if selective contracting is implemented by their health insurer.

H5: Healthier enrolees are more accepting of selective contracting by their health insurer.

H6: Younger enrolees are more accepting of selective contracting by their health insurer.

As explained before, enrolees may perceive a risk in insuring through a health policy with selectively contracted care because they do not know what care they may need in the future and they do not have experience with all the contracted care providers. Since women are more risk averse than men [23], we expect that women are more reluctant to accept selective contracting than men.

H7: Women are more reluctant to accept selective contracting by their health insurer.

## Methods

### Design

This study was conducted in November 2010 among members of a panel of people with health insurance known as the Insurance Panel. This panel was set up in 2006 by the NIVEL (Netherlands Institute for Health Services Research) in cooperation with a large Dutch health insurance company. All members of the panel are insured through this health insurance company. At the time this research was undertaken, the Insurance Panel consisted of 6,732 members who could be approached online via e-mail and 4,759 members who can only be approached through postal surveys. On average, panel members are invited to participate in a survey three times a year. All survey topics are related to health care and health insurance. The panel was registered with the Dutch Data Protection Authority (nr. 1309664). According to Dutch legislation, neither obtaining informed consent nor approval by a medical ethics committee was obligatory for this study. Panel members were free to answer the questions or not.

This survey was conducted online, therefore only the online members of the panel were included. Two reminders were sent to the non-respondents, one seven days, and one 14 days after the initial sending. We received 4,422 completed questionnaires, a response rate of 66%.

### Measures

Variables were measured by single and multi-item measures. All items were measured using a seven point Likert-type scale ranging from completely disagree (1) to completely agree (7). An overview of the multi-item measures is provided in the appendix, see Additional file 1.

### Dependent variable

The dependent variable is the enrolee’s acceptance of selective contracting by their health insurer. Because health insurers in the Netherlands are reluctant to implement selective contracting, enrolees are not yet used to restrictions in their choice of provider. Therefore, the respondents were asked to read an introductory text, where the situation was described in which their health insurer selectively contracts with care providers and that this means that when they go to a not contracted provider they would have to pay a co-payment. Then, respondents were asked in five separate items, to what extent they would agree with their health insurer contracting with only certain hospitals, general practitioners (GPs), physiotherapists, dentists and pharmacies. We asked this question separately for different types of provider, because it is possible that the results differ between the types of provider. Boonen et al. state that the relationship enrolees have with their care provider influences their willingness to switch providers [27]. They find that enrolees have a stronger relationship with their GP than their pharmacy and are therefore more reluctant to accept the selective contracting of GPs than of pharmacies. Although we found slight differences in the acceptance of selective contracting between provider types, factor analysis showed one factor (eigen value 4.04, 1.03 of variance), α = 0.96 (Table 2). The items were merged into one variable by taking the average of the scores on these five items. There were no missing values.

**Table 2:** **Scale Alpha, item means and factor loadings for dependent variable acceptance of selective contracting**^**a**^

**Item**	**Cronbach’s alpha**	**Mean**	**Factor loadings**
To what extent do you agree with your health insurer…			
… Only contracting specific hospitals?	0.96	2.85	0.83
… Only contracting specific general practitioners?		2.48	0.90
… Only contracting specific physiotherapists?		2.74	0.93
… Only contracting specific dentists?		2.62	0.94
… Only contracting specific pharmacies?		2.74	0.89

^a^Items were measured using a seven point Likert-type scale ranging from completely disagree (1) to completely agree (7).

### Independent variables

The independent variables are trust in the health insurer in general (general trust), trust in the purchasing strategy of the health insurer (specific trust) and demographic characteristics including, age, gender and self-reported health status.

Trust was measured in general and, more specifically, in the purchasing strategy of the health insurer in order to explore the influence of trust on the acceptance of selective contracting more in depth. General trust in the health insurer was measured using a validated scale [21], which was translated into Dutch and validated [28]. Trust in the health insurer’s purchasing strategy was measured by asking respondents to indicate in how far they agree with the following statements: (1) I trust my health insurer to choose the best care providers; (2) I trust my health insurer not to compromise on quality in order to keep the price down; and (3) I trust my health insurer to choose the best care for me at the best price. A factor analysis was conducted in order to confirm that the general trust scale and the items that measure specific trust in the purchasing strategy of the health insurer do indeed measure two different constructs. The results of this are presented in Table 3. The factor analysis showed that there are indeed two factors, factor one consisting of 11 general trust items (eigen value 5.13, 0.55 of variance), α = 0.86 and factor two consisting of three specific trust items (eigen value 1.36, 0.46 of variance), α = 0.89. The composite scale general trust was constructed by taking the average of the scores on the items. Specific trust was constructed comparably, using the three specific trust items. There were no missing values. Correlation between these two variables is 0.47, which is acceptable considering both constructs measure trust, although on a different level.

**Table 3:** Scale Alpha’s, item means and factor loadings statistics for independent variables general and specific trust

**Construct**	**Item**^**a, b**^	**Cronbach’s alpha**	**Mean**	**Factor loadings**	
General trust scale	You think the people at your health insurance company are completely honest.	0.86	4.87	0.56	-
	Your health insurer cares more about saving money than about getting you the treatment you need.		4.00	0.41	-
	As far as you know, the people at your health insurance company are very good at what they do.		4.86	0.51	-
	If someone at your health insurance company made a serious mistake, you think they would try to hide it.		4.14	0.53	-
	You feel like you have to double check everything your health insurer does.		4.55	0.61	-
	You worry that private information your health insurer has about you could be used against you.		4.93	0.66	-
	You worry there are a lot of loopholes in what your health insurer covers that you do not know about.		4.42	0.65	-
	You believe your health insurer will pay for everything it is supposed to, even really expensive treatments.		4.54	0.29	-
	If you got really sick, you are afraid your health insurer might try to stop covering you altogether.		5.29	0.59	-
	If you have a question, you think your health insurer will give a straight answer.		5.24	0.61	-
	All in all, you have complete trust in your health insurance company.		4.97	0.64	-
Specific trust in purchasing strategy	I trust my health insurer to choose the best care providers.	0.89	4.61	-	0.81
	I trust my health insurer not to compromise on quality in order to keep the price down.		4.78	-	0.82
	I trust my health insurer to choose the best care for me at the best price.		4.77	-	0.82

^a^Negatively formulated items are reversed.

^b^Items were measured using a seven point Likert-type scale ranging from completely disagree (1) to completely agree (7).

Every year members of the Insurance Panel receive a questionnaire to update their background characteristics. Therefore, age (continuous), gender (0 = male; 1 = female) and self-reported health status (1 poor, 2 moderate, 3 good, 4 very good, and 5 excellent) are known for almost all of the 4,422 respondents to the trust and acceptance questionnaire. Finally, 4,396 respondents could be used for the analyses.

### Statistical analyses

Descriptive statistics were computed to describe the characteristics of the study population.

In order to test the hypotheses a regression model was constructed. We examined the main and the interaction effects. The interaction effects were examined to test the hypotheses that state that the relationship between trust and the acceptance of selective contracting is modified by another variable (H2, H3 and H4). The variables were centred prior to entering the independent continuous variables, general trust, specific trust, self-reported health status, age and their cross-product terms into the regression model. Centring variables means converting each continuous variable to deviation score form, by subtracting a score from respondents’ raw scores, making 0 a meaningful value while preserving the units of the scale. This ensures that the interpretation of effects will occur at a meaningful value of the continuous variable, and it reduces multicollinearity [29]. For age, the rounded mean (56) was subtracted and for general trust, specific trust, and self-reported health scales, the score for 'neutral’ was subtracted.

Interactions that were not significant were removed from the regression model, to facilitate interpretation of the other effects.

## Results

### Descriptives

Table 4 presents descriptive statistics. The mean age of the respondents is 55.6 years ranging from 19 to 90 years and 55.6% of the respondents are male. Less than 14% of the respondents report a poor to moderate health status. The population is older and contains more men compared to the Dutch general population.

**Table 4:** Descriptive statistics of the variables in the model

**Variables**	**Percent/mean**	**N**
Female	44.4%	4422
Age (SD)	55.58 (14.55)	4421
Self-reported health status^a^	3.31	4396
General trust (SD)^b^	4.71 (0.87)	4422
Specific trust (SD)^b^	4.72 (1.48)	4422
Acceptance of selective contracting (SD)^b^	2.69 (1.43)	4422

^a^Measured on a five-point scale ranging from poor (1) to excellent (5).

^b^Items were measured using a seven-point Likert-type scale ranging from completely disagree (1) to completely agree (7).

General trust and specific trust in the health insurer score 4.71 and 4.72 on a scale of one to seven, where seven is the highest and one is the lowest level of trust. These means of 4.71 and 4.72 can be interpreted as moderate general and specific trust. Respondents in general are not very accepting of selective contracting by their health insurer, the average score is 2.69 on a scale of one to seven where seven is most accepting of selective contracting.

### Regression model

Table 5 shows the final regression model. The interaction effect of age and specific trust is significant and was therefore kept in the model. The other hypothesised interaction effects were not significant and therefore removed from the model. Coefficients and standardised regression coefficients are presented to indicate the magnitude of the results found, as well as the level of significance. The main effects will be discussed first, followed by the interaction effect.

**Table 5:** Regression of general trust, specific trust, self-reported health status, age, and gender on acceptance of selective contracting (N = 4396)

	**Coef.**	**Beta**
General trust	0.125	0.076**
Specific trust	0.324	0.335**
Self-reported health	0.048	0.029*
Age	-0.003	-0.029
Female	-0.231	-0.080**
Age*specific trust	0.002	0.032*
Constant	2.443	
Adj. R-square	0.15	

* *p* <0.05; **p <0.001.

*Coef*. Coefficient.

*Beta* Standardised regression coefficient.

### The influence of trust on the acceptance of selective contracting

In line with H1, Table 5 shows that general and specific trust in the health insurer are significantly associated with enrolees’ acceptance of selective contracting by their health insurer. The more trust enrolees have in their health insurer, the more accepting they are of selective contracting by their health insurer. The effect of specific trust is stronger compared to general trust.

### The influence of self-reported health status, age and gender on the acceptance of selective contracting

Table 5 shows that self-reported health status and gender are significantly associated with the acceptance of selective contracting. Age is unrelated to the acceptance of selective contracting. This is in line with H5 and H7; H6 is rejected.

### The interaction effect of age and specific trust on the acceptance of selective contracting

**Figure 1:**
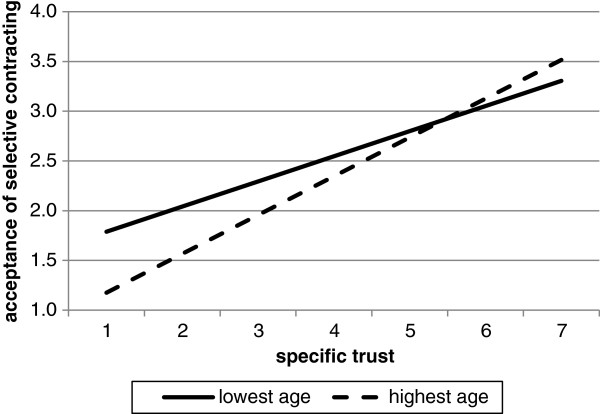
**Association between specific trust and acceptance of selective contracting for people with the lowest age (19) and people with the highest age (90)**^**a**^**.**^a^ For neutral general trust, male, good self- perceived health.

The hypothesised interaction effects between self-reported health and trust and between gender and trust were not significant, therefore H2 en H4 are rejected. Also, the interaction effect between age and general trust was not significant. The results do demonstrate a significant interaction of age and specific trust on the acceptance of selective contracting, partially confirming H3. This means that the relationship between specific trust and acceptance of selective contracting varies by age. In order to determine the nature of the effect, we examined the relationship between specific trust and the acceptance of selective contracting for people with different ages. The coefficient of specific trust presented in Table 5, is indicative of people with a mean age in the model (age = 0, since age is centred^a^). In Figure 1 the relationship between specific trust and acceptance of selective contracting is plotted for people with the highest and the lowest age^b^. For all other ages the plots lie in between the two presented. For both values of age, the relationship between specific trust and the acceptance of selective contracting is positive, meaning that the higher the level of trust, the more accepting enrolees are of selective contracting. However, for the highest age the slope is steeper, that is, the coefficient is higher, which means that the relationship of specific trust and the acceptance of selective contracting is stronger for older people. The effect of specific trust on the acceptance of selective contracting is significant for all ages (*p* < 0.001). This means that specific trust has an effect on the acceptance of selective contracting, no matter the respondent’s age.

The significant interaction effect of specific trust and age also indicates that the effect of age on the acceptance of selective contracting varies with the level of specific trust. The coefficient presented for age in Table 5 is indicative for people who score neutral on specific trust (0, since specific trust is centred). In this situation, the relationship between age and the acceptance of selective contracting is not significant. When plotted for the highest specific trust score, the relationship between age and the acceptance of selective contracting is still not significant. However, when plotted for the lowest specific trust score, the relationship is significant (P < 0.05; coef. = -0.009), showing that younger enrolees are more accepting of selective contracting. This shows that it depends on the level of trust whether or not age has a significant influence on the acceptance of selective contracting. When it does, however, it shows that younger enrolees are more accepting of selective contracting by their health insurer than older enrolees.

## Discussion

Since the shift from a supply-oriented health care system to a demand-oriented one, health insurers have incentives to become prudent buyers of care on behalf of their enrolees. Health insurers are allowed to selectively contract care providers, which is supposed to stimulate competition between care providers and to increase the quality and contain costs in the health care system. However, health insurers are still reluctant to implement selective contracting. Health insurers fear that enrolees lack trust in their health insurer and that enrolees have negative attitudes toward restrictions in their freedom of provider choice. However, is this really the case? In this paper we studied the role of trust on enrolees’ acceptance of selective contracting by their health insurer.

Consistent with our hypotheses, we found that enrolees who have more trust in their health insurer in general, and specifically in their health insurer’s purchasing strategy, are more open to selective contracting by their health insurer. Thus, trust is indeed an important prerequisite for enrolees to accept selective contracting by their health insurer.

The effect of specific trust on the acceptance of selective contracting is stronger for older people than for younger people. This could mean that trust is more important for enrolees who are more likely to need care in the near future. Furthermore, in line with our hypotheses, we found that less healthy and female enrolees are less likely to accept selective contracting by their health insurer than healthier and male enrolees. Also, younger enrolees are more likely to accept selective contracting compared to older enrolees. However, this only applies when people have a low level of trust in the purchasing strategy of their health insurer. Thus, besides trust, gender, self-reported health status and age also have an influence on the acceptance of selective contracting. We must note that even when trust is very high, the acceptance of selective contracting is still relatively low. This means that there are other factors besides trust and respondent characteristics that influence the acceptance of selective contracting by health insurers. One of these factors may be the price of the insurance policy. Insurance policies with restrictions in choice of care provider are usually cheaper, this may positively influence the acceptance of selective contracting.

Contrary to our hypotheses, there were no interaction effects found between trust and self-reported health status, and trust and gender, with regard to the acceptance of selective contracting. This means that the effect of trust on the acceptance of selective contracting by the health insurer does not differ for enrolees with different self-reported health status or gender. The expectation was that less healthy enrolees would need more trust because they need to take a greater risk, since they are more likely to need care now and in the near future. Women, too, would need more trust, because they perceive a greater risk, since they are more risk averse. It is possible that these interaction effects are somehow compensated. This is true when health status and gender affect trust. Because we have this data available we checked for this and found that men and healthier enrolees have more trust in their health insurer. This means that gender and health status partly determine the level of trust while trust has a direct effect on the acceptance of selective contracting.

### Scientific implications

This paper adds to the literature that trust in the health insurer is indeed an important prerequisite for enrolees’ acceptance of selective contracting. Most of the variance in acceptance of selective contracting is however not explained by the model. This means there are more factors besides trust and the characteristics of the enrolees that influence their acceptance of selective contracting by their health insurer. A possible explanation is the importance of autonomy. The self-determination theory states that people have a need for autonomy and fulfilment of this autonomy leads to increased well-being [30]. When the health insurer restricts provider choice, people may feel their autonomy is impaired. This could explain why enrolees do not accept selective contracting by their health insurer. It would therefore be interesting to include autonomy in future research on this subject. Furthermore, we also expect that the reason why enrolees do not accept selective contracting is partly due to the effect of loss aversion. Here, a reference point, usually the *status quo*, determines individual preferences and the lack of benefits in giving up an object being greater than the benefits associated with acquiring it [31,32]. Individuals still experience great difficulty in relinquishing their current situation although it was stated that the health insurer contracts care providers selectively based on quality, price and accessibility. So, how to overcome this status quo bias? It may be possible that status quo bias is non-existent or low when people are already looking for a different health insurance policy. This may be the case when their financial situation or their family composition has changed. For future research, it could therefore be important to include in the model whether or not enrolees are currently considering changing their health plans.

### Practical implications

The results of this study indicate that in practice, when health insurers want to implement selective contracting, it is important that their enrolees trust them, especially the older ones. Although trust may not appear to be very low (4.7 on a scale of 1 to 7), there is still room for improvement. Also, it is known that trust in the health insurer is quite low compared to trust in medical specialists, GPs and hospitals [33]. But how can health insurers increase their enrolees’ trust in them? Literature shows that general trust in a company can be increased by improving elements of technical and functional quality [34]. Functional quality refers to *how* the service is being delivered. For instance, in how responsive the health insurer is to a customer’s complaint and the friendliness of the insurer’s personnel. Technical quality addresses the *what* question and reflects customers’ perceptions of the outcome they receive, such as a timely payment of claims. These aspects are relatively easy for health insurers to invest in. However, this is aimed at increasing general trust in the insurance company, not at specific trust in the way health insurers select care providers. US literature shows that enrolees question the motives of a health insurer that practices selective contracting, because they expect their health insurer to be more interested in making money than in selecting good quality care providers for them [12,13]. Also, having knowledge of the trustee’s behaviour is very important in order to enhance trust [35]. Possibly, more trust in health insurers’ purchasing strategies can be generated when health insurers convince enrolees of their motives for selective contracting. Furthermore, Boonen and Schut [9] state that the availability of objective quality information on care providers can solve the problem of a lack of trust, because such information will help health insurers to select care providers based on objective quality information. It will also help communicate this information to their enrolees. This will reduce uncertainty about the health insurer’s behaviour. However, information from the health insurer still needs to be trusted and credible. When information from the health insurer is not trusted, it may be better if health insurers refer to other, highly trusted, parties to present objective quality information such as a GP or patient organisation.

As mentioned above, even when trust is very high, the acceptance of selective contracting is still relatively low. Therefore, solely increasing trust will probably not be enough to significantly increase the acceptance of selective contracting. Future research is needed to investigate other methods that contribute to increasing acceptance of selective contracting.

The finding that male, healthier and, depending on level of trust, younger enrolees are more likely to accept selective contracting, suggests there is the possibility of risk selection in health plans with selectively contracted care. This is consistent with literature where it was found that younger and healthier people more often choose a restrictive health policy where not all care providers are contracted [24-26]. In practice, in the Netherlands, more men than women are enrolled in a restrictive health policy and most of these enrolees are young and perceive themselves as healthy [36]. This presents a problem given the intended goals of selective contracting, because it is especially important that the people who use most care also accept selective contracting by their health insurer. If not, the financial benefits of channelling patients to selectively contracted care providers cannot be realised. It is very important to find out under which circumstances people who use or need care will choose a health plan with restrictions.

### Limitations

We have only included respondents who are enrolees of one health insurance company. Although this is a large company with enrolees from different insurance labels scattered over rural and urban areas, it is possible that levels of trust or acceptance of selective contracting slightly differ from the total Dutch population. The respondents that participated in this study are older than the general population and include more men. However, this does not affect our regression model results, because all subgroups are of sufficient size to perform the association analyses. Furthermore, we have used an online questionnaire, this could bias results as respondents who have no computer or access to internet could not participate. Usually, these are older adults [37], however, since our panel includes older people filling out online questionnaires, and we have performed association analyses, we do not think this has a significant impact on our results. Additionally, it is likely that the role of trust in the acceptance of selective contracting by the health insurer is not independent of context, this could limit generalizability of our findings. For instance, in the Netherlands, enrolees are not used to selective contracting by health insurers compared to the US where selective contracting is more common. Therefore acceptance of selective contracting may be lower in the Netherlands compared to the US. However, the hypotheses are based on international literature. Therefore, we believe that the relationships we found are also interesting in an international context. Furthermore, we must note that there are no standardised measures available to measure enrolees’ acceptance of selective contracting by their health insurer and to measure trust in the purchasing strategy of their health insurer. Yet, we believe that this study provides a good starting point for developing such measures. In addition, the questions were hypothetical because Dutch citizens are not yet familiar with selective contracting. This could influence the outcomes of our study. Lastly, our data was obtained using a cross-sectional study design and, therefore, cannot provide any information about causal links. However, in this study we looked at a situation that is still uncommon in the Netherlands and not yet very dynamic. This survey could be looked at as a first measurement.

## Conclusions

This study provides insight into factors that influence the acceptance by enrolees of selective contracting by their health insurer. It has confirmed that trust plays an important role in this acceptance. This paper is relevant for health insurers because it provides insight into how enrolees feel about selective contracting. Because hypotheses are based on international literature, the results are relevant not only for health insurers in the Netherlands, but also in other countries with demand-oriented health care systems.

## Endnotes

^a^ This is illustrated by the following regression equations:

$$\begin{array}{l} Y=B0+0.324X\left(\mathrm{specific}\;\mathrm{trust}\right)+-0.003\;Z\left(\mathrm{age}\right)\\ \;+0.002\;\text{XZ}\left(\text{specific}\;\text{trust}*\text{age}\right)\\ Y=B0+0.324X\left(\text{specific}\;\text{trust}\right)+-0.003\;0\left(\text{age}\right)\\ \;+0.002\;X0\left(\text{specific}\;\text{trust}*\text{age}\right)\\ Y=B0+0.324X\left(\text{specific}\;\text{trust}\right) \end{array}$$

^b^ In order to do this, the regression model was run with age centred in alternative ways. First, the model was run with age subtracted by the oldest age (90). The results then showed the constant and coefficient for the oldest respondents. Then, the model was run with age subtracted by the youngest age (19), so the results showed the constant and coefficient for the youngest respondents. With these results the plots were made (Figure 1).

## Competing interests

RB, JJ, SW and PG declare that they have no competing interests. During the data collection EC was part-time (40%) employed by VGZ.

## Authors’ contributions

RB analysed the data and drafted the manuscript. JJ and SW provided expertise in the design of the study, interpreting the results and assisted in drafting the manuscript. EC contributed to the design of the study, data collection as well as revision of the manuscript. PG contributed to the interpretation of the results and revision of the manuscript. All authors have read and approved the final manuscript.

## Pre-publication history

The pre-publication history for this paper can be accessed here:

http://www.biomedcentral.com/1472-6963/13/375/prepub

## Supplementary Material

Additional file 1 Appendix containing an overview of the multi-item measures used in this study. Click here for file
