# Supplementary material for: Acceptance of selective contracting: the role of trust in the health insurer
Source: BMC Health Serv Res. 2013 Oct 2;13:375. doi: 10.1186/1472-6963-13-375 (PMC3850712; doi:10.1186/1472-6963-13-375)
Supplement: Additional file 1 — Appendix containing an overview of the multi-item measures used in this study. [file 1472-6963-13-375-S1.docx]

**Appendix: Overview of multi-item measures**

All items were measured on a 7 point Likert-type scale from completely disagree (1) to completely agree (7).

| **Openness to selective contracting by the health insurer α=0.955**  Introductory text: The health insurer contracts selected care providers based on quality, price and availability. Care providers that are not selected by your health insurer will not be (fully) reimbursed. | | **Mean** |
| --- | --- | --- |
| 1 | To what extent do you agree with your health insurer contracting only certain hospitals? | 2.85 |
| 2 | To what extent do you agree with your health insurer contracting only certain GPs? | 2.48 |
| 3 | To what extent do you agree with your health insurer contracting only certain physiotherapists? | 2.74 |
| 4 | To what extent do you agree with your health insurer contracting only certain dentists? | 2.62 |
| 5 | To what extent do you agree with your health insurer contracting only certain pharmacies? | 2.74 |

| **General trust (Zheng *et al.* 2002; Hendriks *et al*. 2008) α=0.859** | | **Mean** |
| --- | --- | --- |
| 1 | You think the people at your health insurance company are completely honest. | 4.87 |
| 2 | Your health insurer cares more about saving money than about getting you the treatment you need. | 4.00 |
| 3 | As far as you know, the people at your health insurance company are very good at what they do. | 4.86 |
| 4 | If someone at your health insurance company made a serious mistake, you think they would try to hide it. | 3.86 |
| 5 | You feel like you have to double check everything your health insurer does. | 3.45 |
| 6 | You worry that private information your health insurer has about you could be used against you. | 3.07 |
| 7 | You worry there are a lot of loopholes in what your health insurer covers that you don’t know about. | 3.58 |
| 8 | You believe your health insurer will pay for everything it is supposed to, even really expensive treatments. | 4.54 |
| 9 | If you got really sick, you are afraid your health insurer might try to stop covering you altogether. | 2.71 |
| 10 | If you have a question, you think your health insurer will give a straight answer. | 5.24 |
| 11 | All in all, you have complete trust in your health insurance company. | 4.97 |

| **Specific trust α=0.894**  Introductory text: Imagine that your health insurer decides to exclude several care providers from reimbursement. In this case, only the care providers that were selected by your health insurer will be fully reimbursed. If you would want to consult a care provider that has not been selected by your health insurer, you would have to pay an out-of-pocket payment. Your health insurer selects care providers based on quality, price and accessibility. | | **Mean** |
| --- | --- | --- |
| 1 | I trust my health insurer to choose the best care providers. | 4.61 |
| 2 | I trust my health insurer not to compromise on quality in order to keep the price down. | 4.78 |
| 3 | I trust my health insurer to choose the best care for me at the best price. | 4.77 |
